# Hyaluronan regulates sperm-induced inflammatory response by enhancing sperm attachment to bovine endometrial epithelial cells *via* CD44: *in-silico* and *in-vitro* approaches

**DOI:** 10.3389/fendo.2023.1134868

**Published:** 2023-05-10

**Authors:** Mohamed Aboul Ezz, Alireza Mansouri, Ihshan Akthar, Mohamed Samy Yousef, Rasoul Kowsar, Akio Miyamoto

**Affiliations:** ^1^ Global Agromedicine Research Center (GAMRC), Obihiro University of Agriculture and Veterinary Medicine, Obihiro, Japan; ^2^ Department of Theriogenology, Faculty of Veterinary Medicine, Mansoura University, Mansoura, Egypt; ^3^ Department of Theriogenology, Faculty of Veterinary Medicine, Assiut University, Assiut, Egypt; ^4^ Department of Animal Sciences, College of Agriculture, Isfahan University of Technology, Isfahan, Iran

**Keywords:** uterus, sperm, hyaluronan, CD44, TLR2

## Abstract

Recently, we reported that sperm induce cluster of differentiation 44 (CD44) expression and Toll-like receptor 2 (TLR2)-mediated inflammatory response in bovine uterus. In the present study, we hypothesized that the interaction between CD44 of bovine endometrial epithelial cells (BEECs) and hyaluronan (HA) affects sperm attachment and thereby enhancing TLR2-mediated inflammation. To test our hypothesis, at first, *in-silico* approaches were employed to define the binding affinity of HA for CD44 and TLR2. Further, an *in-vitro* experiment using the sperm-BEECs co-culture model was applied to investigate the effect of HA on sperm attachment and inflammatory response. Here, low molecular weight (LMW) HA at different concentrations (0, 0.1, 1, or 10 µg/mL) was incubated with BEECs for 2 h followed by the co-culture without- or with non-capacitated washed sperm (10^6^/ml) for additional 3 h was performed. The present in-silico model clarified that CD44 is a high-affinity receptor for HA. Moreover, TLR2 interactions with HA oligomer (4- and 8-mers) target a different subdomain (h-bonds) compared to TLR2-agonist (PAM3) which targets a central hydrophobic pocket. However, the interaction of LMW HA (32-mers) with TLR2 revealed no stability of HA at any pocket of TLR2. Notably, the immunofluorescence analysis revealed the HA localization in both endometrial stroma and epithelia of *ex-vivo* endometrial explant. Moreover, ELISA showed significant levels of HA in BEECs culture media. Importantly, BEECs pretreatment with HA prior to sperm exposure increased the number of attached sperm to BEECs, and upregulated the transcriptional levels of pro-inflammatory genes (*TNFA, IL-1B, IL-8, and PGES*) in BEECs in response to sperm. However, BEECs treated with HA only (no sperm exposure) did not show any significant effect on the transcript abundance of pro-inflammatory genes when compared to the non-treated BEECs. Altogether, our findings strongly suggest a possible cross-talk between sperm and endometrial epithelial cells *via* HA and HA binding receptors (CD44 and TLR2) to induce a pro-inflammatory response in bovine uterus.

## Introduction

Upon mating or artificial insemination (AI), semen provokes a physiological inflammatory reaction in the female genital tract of humans and animals (1–6). A such inflammatory reaction is characterized by the rapid and transient influx of immune cells, mainly polymorphonuclear cells (PMNs), into the uterine lumen which is critical for not only the removal of dead/excess sperm with associated contaminants (7), but also for preventing the activation of the acquired immune response towards male gametes (sperm) (8).

Bovine uterus has a well-regulated immune response to eliminate bacterial contamination after parturition, tolerate the allogenic sperm and accept semi-allogenic embryos (9). Pattern recognition receptors (PRRs), innate immune cell receptors expressed by the endometrium, have the ability to distinguish “infectious non-self” from “non-infectious self” (9–12). Among PRRs families, Toll-like receptors (TLRs) are involved in the initiation of inflammatory responses to either infections or sterile tissue injuries through recognition of highly conserved molecules called pathogen-associated molecular patterns (PAMPs) or damage-associated molecular patterns (DAMPs), respectively (13). Recently, our research group has demonstrated, *via* employing a series of *in vitro-* (14) and *ex vivo* (15) investigations, that active sperm attach to bovine endometrial epithelial cells (BEECs) and thereby stimulate a pro-inflammatory response through activation of TLR2 signaling pathway. However, which molecule(s) linking sperm to TLR2 pathway in BEECs is still unclear.

Different exogenous or endogenous molecules have been reported as ligands and/or regulator for TLR2 signaling (16); one of these molecules is hyaluronan (HA) (17). HA, a non-sulphated glycosaminoglycan, is normally present in most of mammalian tissues and fluids including those of reproductive system (18). Definitely, HA is secreted in the seminal- (19), oviductal- (20), uterine- (21), and cervical fluids (22) of different species including humans. As well, HA is localized to both granulosa- and cumulus cell layers of the ovarian follicles (23–25). So far, HA has gained a special relevance in the field of reproductive biology due to its participation in numerous physiological events such as ovulation (26), fertilization (27), and cervical ripening prior to labor induction (28). In the last few decades, several reports have linked HA to TLRs activation with a consequent initiation of a pro-inflammatory cascade (29) in a variety of cell types including endothelial cells, epithelial cells, fibroblasts, dendritic cells, as well as macrophages (30–34). In regard to sperm physiology, it has been shown that HA fragments, produced by sperm-released hyaluronidase, activate TLR2/4 signaling pathway with subsequent cytokine/chemokine production in the cumulus cells of cumulus-oocyte complexes (COCs) which is essential for accomplishment of fertilization process (27).

Cluster of differentiation 44 (CD44), a transmembrane protein with multiple isoforms due to its frequent alternative splicing and post-translational modifications, is a major HA receptor (35). CD44 proteins, a class of cell surface adhesion molecules, are ubiquitously expressed throughout the body (35, 36). Other than its well-known function in facilitating cell adhesion and migration, CD44 can mediate numerous cellular pathways *via* recruitment and assembly of signaling proteins (36). Further, CD44 with its ligand HA are involved, either *via* cell-cell interaction or cell-matrix interaction, in promotion of inflammatory process (36, 37). Ligation of CD44 to HA is crucial for leukocytic infiltration (38, 39), T-cell- proliferation and activation (40, 41), as well as cytokines and chemokines production (30, 42–44).

Lately, we have shown that sperm upregulate the gene and protein expression of CD44 adhesion molecule, in both BEECs- and uterine explant models, in the course of sperm-induced uterine inflammation in bovine (45). Altogether, HA could be a good candidate to act as a bridging ligand between the sperm cells from one side and CD44 of BEECs from the other side for regulating sperm attachment, and TLR2-mediated inflammation induced by the sperm. To test the above hypothesis, we first performed in-silico approaches to detect the binding affinity of HA molecules for CD44 and TLR2. Then, we determined the existence of HA in the uterine environment *via* immunofluorescence and enzyme-linked immunosorbent assay (ELISA). Further, sperm-BEECs *in-vitro* co-culture model was applied to investigate the impact of BEECs pretreatment with exogenous HA on sperm attachment and subsequent immune response.

## Material and methods

### *In-silico* investigations

#### Phase I: preparation of CD44, TLR2 and HA molecules

For conducting the in-silico investigations, human HA-binding proteins [i.e., CD44 (PDB ID: 1UUH) and TLR2 glycoprotein (PDB ID: 2Z7X)] were applied; the sequence identity between human- and bovine HA binding proteins is 92.5 and 72.27 for CD44 antigen and TLR2 extracellular domain, respectively. Additionally, template-based modeling (46) showed that both proteins in human and bovine species have similar folding, as the template modelling (TM) scores of 0.8 and 0.86 were obtained for TLR2 and CD44, respectively (**Supplementary Figure 1**).

The three-dimensional (3-D) structure of HA with 4-, 8- and 32-mers were generated as previously described (47, 48). For optimizing of all the above molecules, molecular dynamics (MD) simulations were operated (49–52).

#### Phase II: docking simulation of HA to CD44 and TLR2

The optimized HA4, HA8 and HA32 structures, obtained from phase I, were used for docking studies by AutoDock VINA (v.1.2.0) (53). In this study, HA structures without any routable bond were considered to be ligands whereas the binding proteins as rigid entities were considered to be receptors. For docking calculation, the main binding sites of both proteins were selected as the grid box. To compare the interaction of HA molecules to the main binding site of TLR2, the complex of TLR2/1-Pam3CSK4 (PDB ID: 2Z7X) was used to perform MD simulation for 150 ns. PamCSK4 (PAM3) is a synthetic triacylated lipopeptide (LP) and specific agonist for TLR2 which mimics the acylated amino terminus of bacterial LPs. The ligand orientation of HA to the main binding site of receptor with the lowest binding energy were selected for further analysis and MD simulation.

#### Phase III: Molecular dynamics (MD) simulation of binding proteins/HA complexes

MD simulation (during 150 ns) and molecular mechanics Poisson–Boltzmann surface area (MM-PBSA) methods were used to calculate and predict the binding free energy (BFE) (**Supplementary Data**) (54). Radial distribution function (RDF) and center of mass (COM) were calculated to describe the atomic interaction between HA and hyaluronan binding proteins (55, 56).

### *In-vitro* investigations

#### Uterine samplings

The uterine samples used for immunostaining as well as isolation and culture of BEECs were brought from a local slaughterhouse (Hokkaido Livestock, Doto Plant Tokachi Factory; Obihiro, Hokkaido, Japan). Simply, the bovine uterine horns (contra-lateral to mature follicle) were carefully opened and grossly examined to be free from any abnormalities. Only healthy uterine horns (ipsi-lateral to mature follicle) from the pre-ovulatory phase (Days 19-22) were collected, immersed in physiological saline with antibiotics [1% penicillin-streptomycin (Gibco, Grand Island, NY, USA) and 1% amphotericin B (Gibco)], and then transported to the laboratory within 1-1.5 h on ice. Of note, the phase of estrous cycle was determined on the basis of corpus luteum appearance, size, and color as well as the follicular diameter (57).

#### HA immunostaining

The endometrial sections used for immunostaining were prepared as previously described (15). In brief, the endometrial tissue explants were dissected from the glandular (intercaruncular) endometrial regions. Then, the explants were fixed in formalin, embedded in paraffin, and 4 µm thick endometrial sections were prepared. HA localization to the bovine endometrium was determined by immunofluorescence staining using biotinylated HA-binding protein (bHABP) according to (58, 59) protocols with minor modifications. Briefly, the endometrial sections were incubated with 1% BSA for 30 min at room temperature (RT) to block the non-specific binding sites. Afterwards, the sections were incubated with bHABP (5 µg/mL, #385911, Calibiochem) overnight at 4 °C followed by labeling with Streptavidin-Alexa Fluor conjugate (2 µg/ml, #S11223, Thermo Fisher Scientific) for 1h at RT. The sections were then mounted using VECTASHIELD mounting medium with DAPI (H-1200, Vector Laboratories). Negative controls were performed by pre-digesting sections with hyaluronidase (#H3506, Sigma Aldrich) to ensure the specificity of the reaction. The fluorescence signal was captured using an all-in-one fluorescence microscope (Keyence, BZ-X800) using BZ-X GFP (OP-87763)-, and BZ-X DAPI (OP-87762) filters set for the green- and blue wavelengths, respectively. Exposure time was constant for all sections including negative control. The experiment was repeated three times using endometrial explants from three different uteri.

#### BEECs isolation and culture

Following the previously described protocols (60, 61) with minor modification, BEECs were isolated (from the obtained uterine horns) and then cultured. In brief, the epithelial cells were detached and suspended in Dulbecco’s Modified Eagle Medium: Nutrient Mixture F12 (DMEM/F12) (Gibco) supplemented with 22 mM NaHCO3 (Sigma-Aldrich, St. Louis, MO, USA), 0.1% gentamicin (Sigma-Aldrich), 1% amphotericin B (Gibco), and 10% heat-inactivated fetal calf serum (FCS) (Bio Whittaker, Walkersville, MD, USA). Then, cells were seeded in 25 cm^2^ culture flasks (Nalge Nunc International, Roskilde, Denmark), and cultured at 38.5˚C in a humidified atmosphere of 5% CO2 in air. Upon reaching 70-80% (sub-confluence), the cells were passaged, trypsinized, and re-seeded (at 1 x 10^5^ cells/ml) in 1.5 ml/well culture medium (DMEM/F12, 22 mM NaHCO3, 0.1% gentamicin, 1% amphotericin, and 5% FCS) in 12 well plates (Nalge Nunc International) until sub-confluence. These plated cells were exposed to estradiol-17β (E2; 50 pg/ml) and progesterone (P4; 1 ng/ml) throughout the whole culture period to simulate the pre-ovulatory phase *in situ* (62, 63). The purity of epithelial cells in our model (>98%) was ensured *via* immunofluorescence labelling with a monoclonal antibody against cytokeratin (anti-cytokeratin 8 + 18; ab53280, Abcam, Tokyo, Japan) (64).

#### Sperm preparation

For getting sperm, frozen 0.5 ml semen straws were obtained from three highly fertile Holstein bulls belonging to Genetics Hokkaido Association, Hokkaido, Japan. Frozen semen straws from three bulls were thawed in a water bath at 38.5°C for 30 sec, pooled, and washed 3 times in a Tyrode’s albumin, lactate, and pyruvate medium (Sp-TALP) (65). The progressive motility of the recovered sperm, as assessed by visual examination using a light microscope equipped with a stage warmer, was around 50%.

#### Co-culture of BEECs with sperm

Sub-confluent BEECs monolayers in 12-well plates were incubated in 1 ml/well culture medium (DMEM/F12, 22 mM NaHCO_3_, 0.1% gentamicin, 1% amphotericin, 0.1% FCS as well as E_2_ and P_4_ at the above-mentioned concentrations) supplemented with LMW HA (Select-HATM Hyaluronan; mol wt 25-75 kDa, #S0326, Sigma) at different concentrations (0, 0.1, 1, or 10 µg/mL) for 2 h followed by the co-culture without- or with non-capacitated washed sperm (10^6^/ml) for additional 3 h (64). This experiment was repeated five times using epithelial cells from five different uteri.

#### Quantification of HA levels

At the end of co-culture period, BEECs-conditioned media were collected, centrifuged twice at 1000 g for 10 min at 4 °C and kept at -80 °C for HA quantification. Commercially available HA ELISA kit (DHYALO, R&D Systems, Minneapolis, MN) was used for determination of HA concentrations in BEECs-conditioned media. A 50 µL aliquot of each sample was analyzed according to the manufacturer’s instructions. All samples were run in duplicate. Optical density (OD) readings were performed at 450 nm. Control group was run to determine the baseline concentration of HA in DMEM culture media. This experiment was repeated four times using epithelial cells from four different uteri.

#### Determination of the number of attached sperm

To determine the number of attached sperm, BEECs monolayers in 12-well plates (1 ml/well culture medium without- or with HA) were exposed to washed sperm (10^6^/ml) for 30 min followed by video capturing using a light microscope (at 200 x magnification) equipped with a stage warmer and digital camera connected to EOS utility software^®^ (Canon U.S.A., Inc.); the focus was adjusted during video capturing to visualize all attached sperm. Five random fields were captured per each group. Of note, videos of the different groups within the same experiments were captured under the same field area and video setting (15).

On the other hand, to determine the number of sperm remained attached, BEECs monolayers in 12-well plates (2 ml/well culture medium without- or with HA) were exposed to 10^6^ sperm/well for 3 h. Afterwards, the upper 1.5 ml media were very gently aspirated, centrifuged at 1000 x g, and the pellet was then re-suspended and counted independently by two investigators using a hemocytometer. To calculate the number of sperm remained attached to BEECs, we subtracted the number of detached sperm (dead and/or floating) from the total number of sperm (10^6^/well) (64).

#### RNA extraction, cDNA synthesis, and quantitative real-time PCR

At the end of sperm-BEECs co-culture, RNA was extracted from BEECs using Trizol reagent (Invitrogen, Carlsbad, CA, USA), quantified by means of a NanoDrop Spectrophotometer 2000c (Thermo Scientific, Waltham, MA, USA), and then pure RNA samples (i.e., A260/A280 ratio were between 1.8 and 2.0) were kept in RNA storage solution (Ambion, Austin, TX, USA) at -80°C till cDNA synthesis (66).

The synthesis of cDNA was performed as previously decribed (67) with minor modifications. First, the extracted RNA was subjected to a DNase treatment step using RQ1 RNase-Free DNase kit (Promega, Madison, WI, USA) to remove residual genomic DNA as well as other contaminants. At such step, 1 μg of extracted RNA was incubated with the first mixure [1 μl of RQ1 RNase-free DNase 10X Reaction Buffer, 2 μl of RQ1 RNase-free DNase (1 unit/μl), and Nuclease-free water (Invitrogen, Carlsbad, CA, USA) to a total volume of 10 μl] for 30 min at 37°C in a thermal cycler (Eppendorf, Hamburg, Germany), then 1 μl of the RQ1 DNase Stop solution was added for 10 min at 65°C to stop this reaction. Afterwards, the first-strand cDNA was produced *via* SuperScript II Reverse Transcriptase kit (Invitrogen) according to the manufacturer instructions. In brief, the DNase-treated RNA was incubated with the second mixture [1.5 μl of 3 μg/μl random primer, 1.5 μl of 10 mM PCR Nucleotide Mix (dNTP) (Roche Diagnostics, Indianapolis, IN, USA) and Nuclease-free water to a total volume of 18 μl] at 65°C for 5 min. Then, the third mixture [6 μl of 5X First-Strand Buffer, 3 μl of 0.1M dithiothreitol and 1.5 μl of 40 units/μl Ribonuclease Inhibitor Recombinant (Toyobo, Osaka, Japan)] was added per each tube and incubated at 42°C for 2 min. Finally, 0.2 μl of 200 units/μl SuperScript II Reverse Transcriptase was added and the thermal cycler was programmed at 25°C for 10 min, 42°C for 50 min and then 70°C for 15 min. The synthesized cDNA was stored at -30°C.

The transcriptional levels of TLR2, Tumor necrosis factor (TNF) alpha (TNFA), Interleukin (IL)-1 beta (IL-1B), IL-8, and Prostaglandin E synthase (PGES) were detected *via* a quantitative real-time polymerase chain reaction (PCR) by means of an iQ5 real-time PCR detection system (Bio-Rad Laboratories, Tokyo, Japan). To clarify, a total 10 μl reaction mix [i.e., 2 μl/sample synthesized cDNA, 5 μl of QuantiTect SYBR Green PCR Master Mix (QIAGEN, Hilden, Germany), 0.2 μl of the targeted primer pairs (listed in **Supplementary Table 1**), and 2.8 μl nuclease-free water (Invitrogen)] was run in amplification program with an initial denaturation step at 95°C for 15 min, followed by 40 cycles of denaturation at 95°C for 15 sec, annealing at 51°C for 30 sec, extension at 72°C for 20 sec. A negative control (reactions containing nuclease-free water or non-reverse transcribed RNA) were involved in each run. It should be emphasized that we here used Primer Express Software v3.0.1 (Thermo Scientific) to design the used primers’ pairs. The calculated cycle threshold (Ct) values were normalized against ACTB (β-actin); no significant variances were detected in β-actin mRNA expression among the different treatments. The delta-delta Ct (2^–ΔΔCt^) method was applied to estimate the fold change between the different samples (64, 67, 68).

### Statistical analysis

Each experiment was repeated at least three times using epithelial cells from 3–4 different uteri. In each uterus, 3 replicates were performed (3 wells per treatment per experiment) and data are presented as mean ± standard error of the mean (SEM). Student’s t-test was applied to compare the data between two groups, while one-way ANOVA followed by Tukey’s multiple comparisons test was used for more than two groups. The results were considered statistically significant at P< 0.05 and P< 0.0001.

## Results

### *In-silico* investigations

The determination of initial structure of HA-receptors complex for MD simulations and energy analysis have been performed using docking simulations. The top three binding orientations of HA with main binding site of proteins with estimated BFEs through Autodock VINA were detected (**Supplementary Figure 2**). HA showed a potential interaction with CD44 through crystallographic mode (residues presented in the main binding site like Ile 96, Tyr 79, Ser 109, etc.). HA was not able to interact with the internal pocket of TLR2 and the residues involved in TLR2-HA interaction (mainly hydrogen bonds) were different form TLR2-PAM3 interaction (hydrophobic bonds) through Ligplot analysis (69) (**Supplementary Figure 3**).

**Figure 1:**
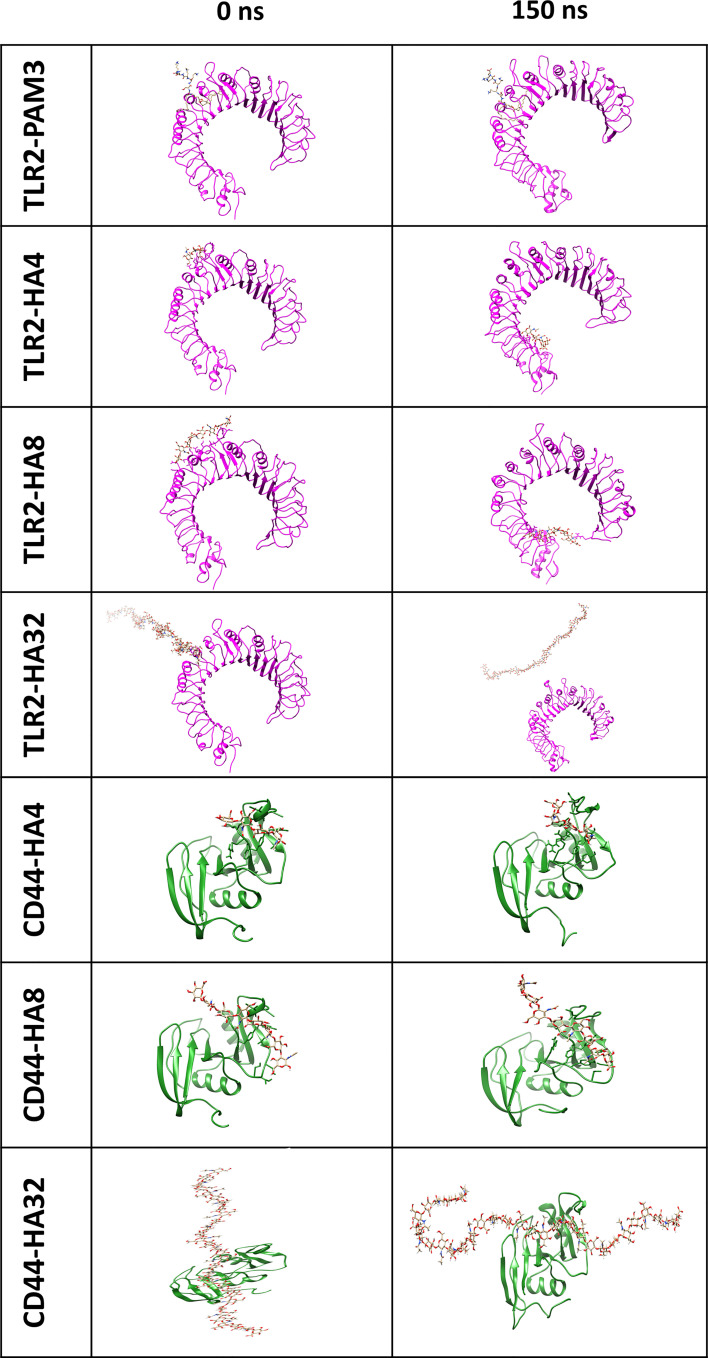
The first and final snapshots of HA/TLR2 and HA/CD44 complexes (with representation of magenta and green ribbon for TLR2 and CD44, respectively and stick for HA) after 0 and 150 ns simulation time. Note that, HA could not stay and bind to the main binding site of TLR2, as PAM3 during 150 MD simulation time, however, HA oligomers (HA4 and HA8) moved and stayed in peripheral domain of TLR2. Concerning CD44, HA indicated a strong affinity since the location of HA did not change the after applying 150 ns simulation time.

**Figure 2:**
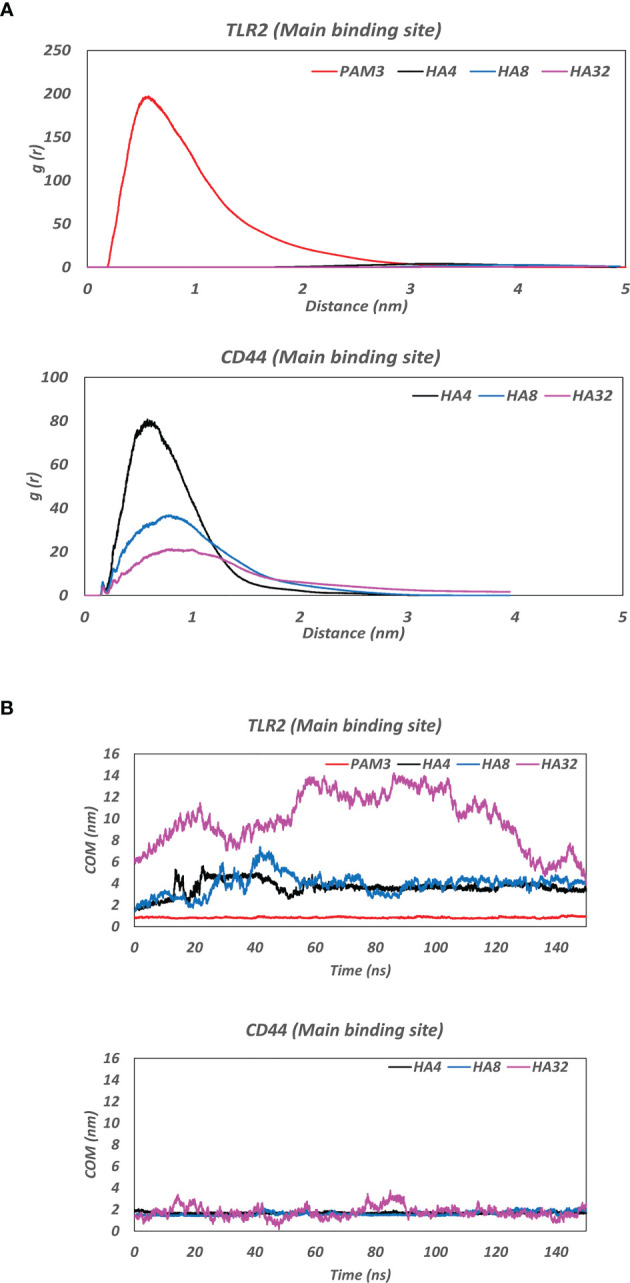
**(A)** Radial distribution function (RDF) of the complex of HA and the main binding site of receptors (TLR2 and CD44) during the last 10 ns MD simulation. Note the clear peak for PAM3-TLR2 at ∼ 5Å and for HAs-CD44 at ~ 7Å which indicating a high affinity of ligands (PAM3 and HA) to their receptors (TLR2 and CD44, respectively). **(B)** The average of center of mass (COM) distance between HA and the main binding site of receptors (TLR2 and CD44) during 150ns MD simulation. Note the fluctuations between TLR2 before 60 ns MD. COM distance between both HAs and CD44 was nearly constant around 2nm throughout the 150 ns MD.

All HA polymers (HA4, HA8 and HA32) could not stay in the vicinity of the main binding site of TLR2 during MD simulation (**Figure 1**). However, CD44 has the main binding domain for all HAs. Moreover, the BFE of HAs-CD44 proposed the high affinity of HA to this receptor (-723.4, -1272.5 and -1680.6 kJ/mol for HA4-CD44, HA8-CD44 and HA32-CD44, receptively). Additionally, HA with higher number of monomer (HA32) showed stronger interaction to the main binding site of CD44 comparing to HA4 or HA8, since more residues in the binding pocket involved in the h-bond interaction (**Supplementary Figure 4**). RDF (calculated during the last 10 ns simulation time) of the complex of HA in the main binding site of receptors was showed in **Figure 2A**. RDF calculated for PAM3 and HAs induced individual peaks for PAM3 at TLR2 main binding distance of ∼ 5Å. As for HA4 and HA8 in HA-TLR2 complexes, the peaks of RDF were appeared at distance of ~ 30 Å. Regarding CD44 crystallographic mode, the RDF of HA4, HA8 and HA32 has peaks at ~ 7Å which indicating a high tendency of HA to CD44.

**Figure 2B** illustrates the distance averages of COM between HAs and main binding sites of receptors. Regarding the main binding site of TLR2, there were fluctuations between 3 and 4 nm for HA4 and HA8 and became stable after 60 ns, as they moved and stabilized into another subdomain in TLR2. However, as for HA32, the ligand failed to interact with any pockets of TLR2. Looking at CD44, the fluctuation of COM distance was not changed during 150 simulation time (the amplitude of the oscillations is between around 1.5 and 2 nm for all HA). HA tried to preserve the initial distance (positions obtained from docking simulation) during 150 ns MD simulation time.

### Immunostaining and ELISA experiments

**Figure 3:**
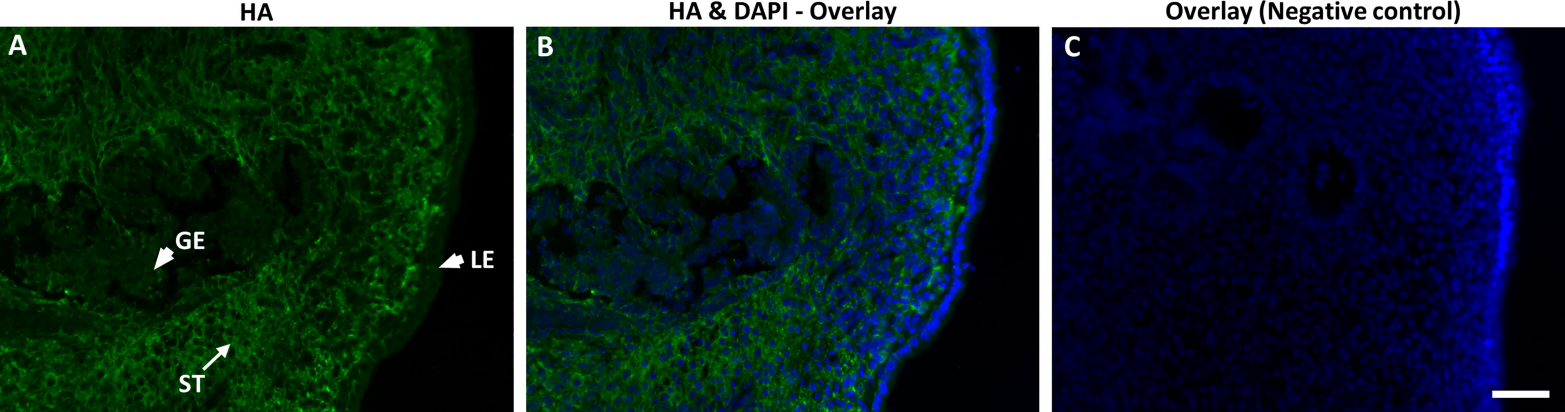
Immunofluorescence localization of hyaluronan (HA) using biotinylated HA-binding protein (bHABP) in bovine endometrium at pre-ovulatory stage visualized using streptavidin-Alexa Fluor conjugate. **(A, B)** HA shown as green staining while **(B, C)** nuclei stained blue with DAPI. Arrows indicate strong expression of HA in stroma (ST), while arrowheads indicate weak expression in luminal (LE) and glandular epithelium (GE). **(C)** The negative control sections pre-incubated with hyaluronidase shows no staining. Bar = 50µm.

To ensure the localization of HA to bovine endometrium, the endometrial sections were subjected to immunostaining using bHABP. Indeed, HA was localized to bovine endometrium. Although a higher expression was observed in the endometrial stroma, HA was also expressed by luminal- and glandular epithelia of bovine endometrium (**Figure 3**). Negative control sections treated with hyaluronidase showed no staining, demonstrating the specificity of the staining.

Concurrently, the conditioned media *in-vitro* produced *via* incubating the culture media with BEECs monolayers showed detectable levels of HA with an average of 16.05 ± 2.33 ng/ml.

### Cell culture and gene expression experiments

**Figure 4:**
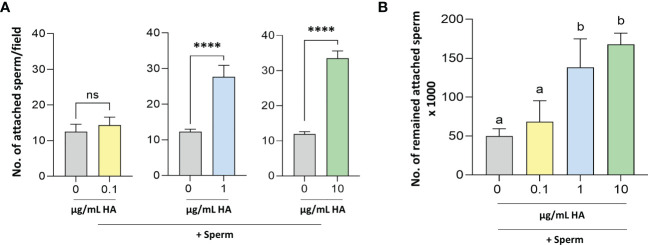
Determination of the number of **(A)** attached and **(B)** remained attached sperm to BEECs pretreated without- with HA. Sperm cells at 10^6^/ml Sp-TALP were exposed to HA (at 0, 0.1, 1, or 10 µg/mL) for 0, 30, 60, and 120 min. Data are presented as mean ± SEM of 3 independent experiments using epithelial cells from 3 different uteri (3 wells per treatment per experiment). Different letters denote a significant variance (*P*<0.05) between the different groups when compared with One-way ANOVA followed by Tukey’s multiple comparisons test, while asterisks denote a significant variance (**** means *P*<0.0001) between the defined groups when compared using Student’s t-test.

Accordingly, it was essential to elucidate the possible role(s) of HA in sperm-BEECs crosstalk. BEECs monolayers were enriched with different concentrations of HA (0, 0.1, 1, or 10 µg/mL) for 2 h prior to the co-culture with sperm for further 3 h. At a glance, we observed that the number of attached sperm was significantly higher in BEECs treated with HA (at either 1 or 10 µg/mL) than in the non-treated BEECs (**Figure 4A**). To confirm such observation, we quantified the number of sperm remained attached to BEECs at the end of co-culture period (3 h). Likewise, BEECs pretreatment with HA dose-dependently increased the number of remained attached sperm (**Figure 4B**).

To exclude the possibility that HA could adversely affect sperm dynamics, sperm cells at 10^6^/ml were exposed to HA at the above concentrations for 2 h. Then, the progressive motility of recovered sperm was assessed at 0-, 30-, 60-, and 120-min post-exposure. Importantly, HA did not affect sperm progressive motility over the exposure period (**Supplementary Figure 5**).

**Figure 5:**
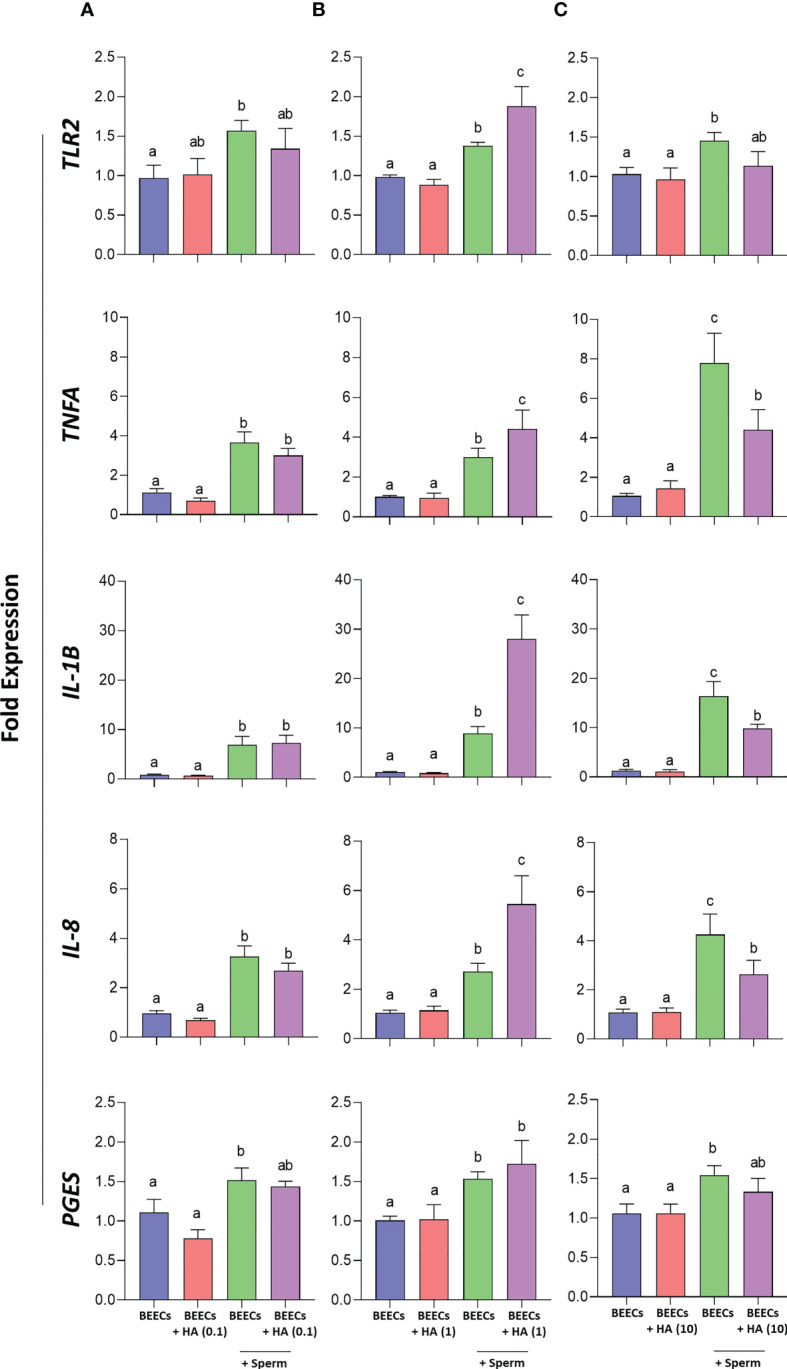
Effect of BEECs pretreatment with HA on sperm- induced inflammation. **(A–C)** BEECs monolayers were incubated without- or with HA (at 0.1, 1, or 10 µg/mL) for 2 h prior to the co-culture with 10^6^ sperm/mL for 3 h. Then, mRNA expressions of *TLR2, TNFA, IL-1B, IL-8*, and *PGES* were quantified in BEECs *via* a real-time PCR. Data are presented as mean ± SEM of 3 independent experiments using epithelial cells from 3 different uteri (3 wells per treatment per experiment). Different letters denote a significant variance (*P*<0.05) between the different groups when compared with One-way ANOVA followed by Tukey’s multiple comparisons test.

Afterwards, it was crucial to investigate the effect of BEECs pretreatment with HA on sperm-induced inflammation. Therefore, BEECs monolayers were co-incubated with HA at either 0, 0.1, 1, or 10 µg/mL for 2 h followed by the co-culture with sperm for additional 3 h. Then, mRNA expressions of *TLR2*, pro-inflammatory- cytokines (*TNFA* and *IL-1B*) and chemokines (*IL-8*) as well as *PGES* were quantified in BEECs *via* a real-time PCR. Our data showed that the pre-incubation of BEECs with HA at lower concentration (0.1 µg/mL) has no effect on *TLR2, TNFA, IL-1B, IL-8*, and *PGES* mRNA expressions in BEECs triggered with sperm (**Figure 5A**), while HA at medium concentration (1 µg/mL) increases the stimulatory effect of sperm on the abundances of *TLR2, TNFA, IL-1B, IL-8*, and *PGES* transcripts in BEECs (**Figure 5B**). However, we recorded lower transcriptional levels of the aforementioned genes upon sperm co-culture with BEECs treated with HA at higher concentration (10 µg/mL) when compared to the non-treated BEECs (**Figure 5C**).

## Discussion

In support of our previous investigations concerning sperm-maternal immunological crosstalk (14, 15, 45, 64, 67, 70, 71), the present study using *in-silico* analyses combined with *in-vitro* co-culture model of sperm with endometrial epithelial cells provides several lines of evidence that HA, primarily through CD44 interaction, has the capacity to facilitate sperm attachment to the endometrial epithelia and thereby triggering sperm-induced inflammatory response in bovine uterus *via* TLR2 signaling transduction.

HA, the most abundant glycosaminoglycans in female reproductive organs (18), interact with CD44 but could not interact with the main binding site of TLR2. The present study showed that HA has no affinity to the main binding site of TLR2, compared with PAM3 during MD simulation time. Notably, the main binding site of TLR2 is the internal hydrophobic channel, which highly attract lipoprotein, such as PAM3 (72).

According to the Ligplot result, the main bond types for HA interaction were hydrogen bonds (h-bonds). For instance, HA create h-bonds with residues of CD44 (Arg 41, Tyr 42 etc.) to interact strongly. TLR2 presented h-bonds (such as Arg 508, Arg 447 and Arg 486) in a peripheral subdomain, away from the main pocket, which involved in the HA-TLR2 interaction (for 4- and 8-mers HA). It is obvious that polar and electricity charged amino acids play an important role in HA binding affinity. CD44 has three topographically main binding for HA, however HA has the high potential affinity only to crystallographic mode (47, 73). In harmony to previous literatures (47, 73), our computational analysis confirmed the strong interaction between crystallographic binding mode of CD44 with all HAs (BFE: -723.4, -1272.5 and -1680.6 kJ/mol for HA4, HA8 and HA32 respectively). Hence, our *in-silico* investigations strongly suggest that HA has higher binding affinity to the main binding site of CD44 than TLR2.

Based on the above computational analyses, it was fundamental to confirm the existence of HA in bovine uterus. Importantly, the immunohistochemical detection of HA in the pre-ovulatory uterus showed its localization to both endometrial stroma and epithelia. The staining revealed that HA were strongly localized within the stroma and weakly expressed by luminal and glandular epithelium of the endometrium. Similar observations (i.e., intense expression in stroma) were reported in other species such as in ovine (74) and mouse (21) endometrium. In ovine species, HA was expressed strongly in the follicular stage endometrium compared to luteal stage (74). In bovine oviducts, HA was strongly localized to the stroma of the oviductal villi and no expression was observed in the luminal epithelium (59). The observed lower expression of HA in endometrial epithelium may be due to the release of luminal and glandular epithelial HA into the uterine cavity. It is likely that, the lower level of epithelial HA would favor a diffusion from stroma to epithelium which could lead to a dynamic releasing of HA from stroma to the uterine lumen. In support of this, the present ELISA result demonstrated that BEECs have the ability to release detectable levels of HA into the culture media over the co-incubation period.

Accordingly, we next aimed to investigate the effect of BEECs enrichment with HA prior to the co-culture with sperm on the subsequent sperm-BEECs interaction. Interestingly, BEECs pre-enrichment with HA dose-dependently increased the number of sperm attached to BEECs; such phenomenon could be referred to HA-CD44 interaction. In light of our *in-silico* investigations, HA has a much stronger binding affinity to CD44 than TLR2. As well, we have previously reported that CD44 adhesion molecule plays a principal role in sperm attachment to the endometrial epithelia in bovine uterus; the addition of anti-CD44 neutralizing antibody negatively impacted sperm-BEECs interaction (45). Besides, it has been reported that sperm cells could express CD44 (75). Altogether, we speculate that the exogenous HA added to BEECs culture media prior to sperm exposure could act as bridging ligand between sperm from one side and CD44 of BEECs from the other side. Such model has been extensively studied in leukocyte trafficking. Namely, leukocytic infiltration from the bloodstream into inflamed tissues or organs requires binding interactions between adhesion molecules on leukocytes from one side and endothelial cells from the other side; HA-CD44 interaction has been implicated in regulation of rolling-, adhesion- as well as invasion processes ending with leukocytic infiltration (36).

Since sperm attachment to the endometrial epithelia is a prerequisite for promotion of a pro-inflammatory response in bovine uterus *via* activation of TLR2 signaling pathway (14, 45, 64, 70), it was necessary to determine whether enhancing sperm attachment by the aid of BEECs pretreatment with HA could stimulate a much stronger inflammatory response in BEECs. Strikingly, our PCR data showed that BEECs pretreatment with medium HA concentration (i.e., 1 µg/mL) upregulates mRNA expressions *TLR2*, pro-inflammatory- cytokines (*TNFA* and *IL-1B*) and chemokines (*IL-8*) as well as prostaglandins E synthesis (*PGES*). However, higher exogenous HA concentrations (i.e., 10 µg/mL) added to BEECs prior to exposure to sperm weakened sperm-triggered inflammation in BEECs.

In the past few decades, HA was considered as inert constituent of extracellular matrix, but it is currently categorized as “dynamic” molecule with a continuous turnover to HA molecules of various sizes: high molecular weight (HMW) HA, low molecular weight (LMW) HA as well as oligosaccharides. Under the physiological conditions, HMW HA primarily contributes to tissue integrity (76, 77). Upon tissue injury, HMW HA molecules are rapidly degraded into LMW HA molecules, referred as HA fragments. *Via* binding with HA receptors, mainly CD44 adhesion molecule (37), these HA fragments have the capacity to activate a pro-inflammatory response with a subsequent transcription of pro-inflammatory genes (29) such as cytokines (TNFA, IL-8, and IL-12), chemokines [macrophage inflammatory protein (MIP), keratinocyte chemoattractant (KC), macrophage chemoattractant protein-1 (MCP-1), and IFN induced protein-10], matrix-modifying enzymes (MMEs), inducible nitric oxide synthase (iNOS), as well as plasminogen activator inhibitor (30, 42, 44, 78, 79). Concurrent with their capability to induce inflammation through CD44 interaction, HA fragments can signal through a TLR-mediated pathway. Using various murine models, it has been established that HA fragments stimulate cytokine and chemokine production through activation of TLR2/4 signaling pathway in dendritic cells (80), macrophages (81), as well as COCs (27).

On the other hand, it has been shown that CD44 plays a role in stimulating TLR2 downstream targets with a subsequent progression of osteoarthritis as evidenced by the upregulation of NFκB-, IL-1B- as well as TNFA gene expression in human macrophages. This study demonstrated that a reduction in CD44 levels in macrophages, *via* using CD44- specific antibody or knockdown, prior to TLR2 activation downregulates NF-κB transcription and thereby lowers proinflammatory cytokines’ (IL-1B and TNFA) production. In addition, they reported that pretreatment of human macrophages with higher doses of HA (100, 250, or 500 µg/mL) dose-dependently inhibits their pro-inflammatory response upon TLR2 ligation (82). Based on the latter finding, we can conclude that BEECs treatment with a higher dose of HA (10 µg/mL) prior to sperm exposure could reduce CD44-meditated TLR2 activation and thereby diminish sperm-induced inflammation in BEECs.

Importantly, it has been shown that both TLR2 and CD44 molecules can function as coreceptors after stimulation of immune cells with TLR2 agonist (Zymosan) (83). Moreover, HA can form a triple complex with CD44 and TLR2 to promote the invasiveness and pro-inflammatory environment in cancer cells (84). Therefore, it seems that sperm-uterine immune-crosstalk could be regulated by CD44 and TLR2 under the effect of HA. However further investigations are necessary to clarify this phenomenon. Moreover, additional studies are required to identify the post-translational modification (PTMs) and how polymorphism affect TLR2 response toward different ligands in bovine species.

**Figure 6:**
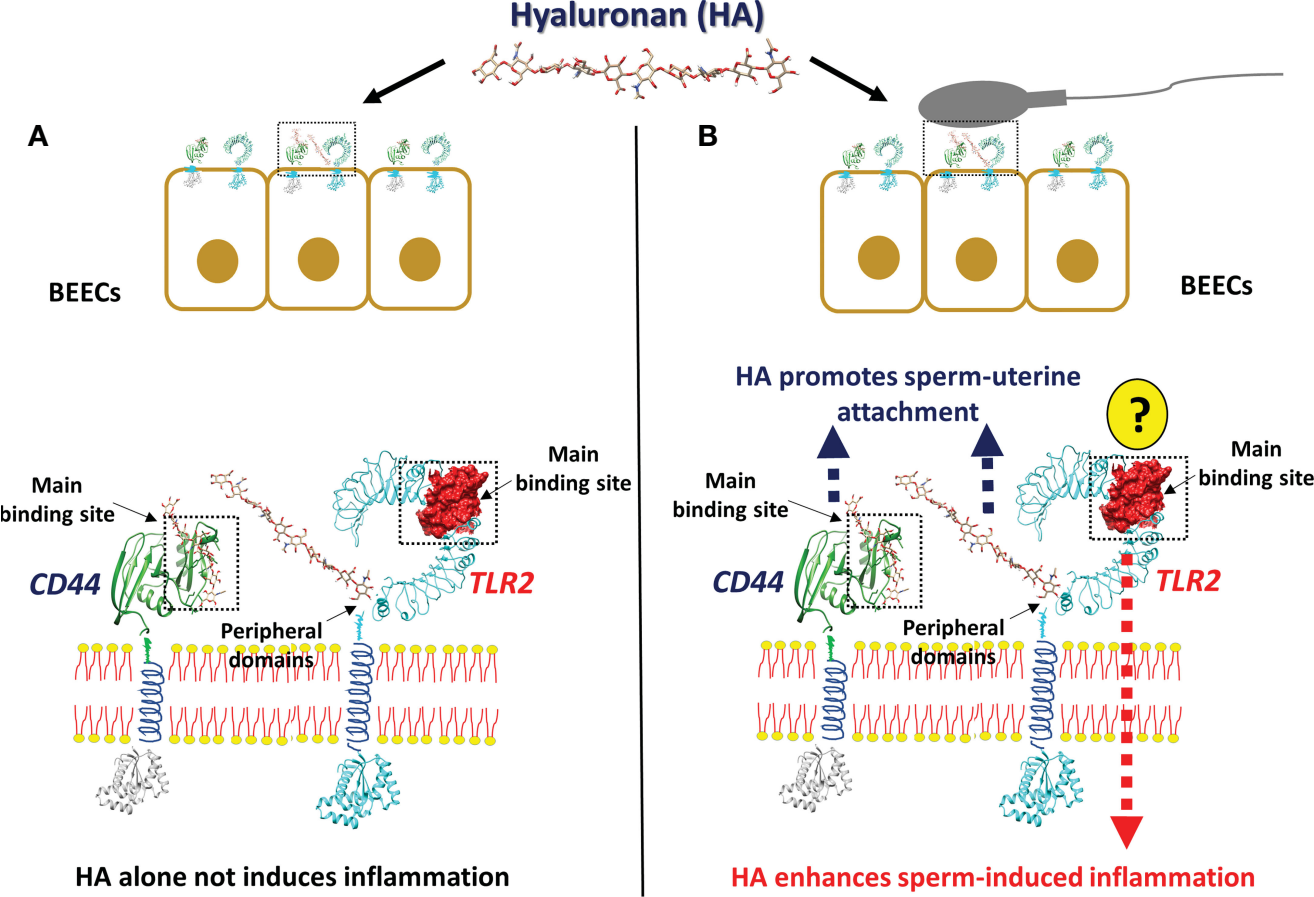
The working hypothesis of the impact of Hyaluronan (HA) on sperm-induced inflammation in BEECs. The anticipated model indicating that, stimulation of BEECs with the most abundant endogenous ligands and molecules in bovine uterine lumen HA, prior to sperm co-culture, accelerate sperm attachment and subsequent inflammation, due to the strong interaction between HA and its receptors. **(A)** HA alone is unable to induce inflammation, since it is unable to interact with the main binding site of TLR2 which is responsible for the triggering of inflammation. **(B)** In the presence of sperm, HA could have an interaction with the main binding site of CD44 and with the peripheral domains of TLR2, which is involved in sperm attachment. After the sperm attachment, the unknown molecules (?) from sperm side should trigger the main binding site of TLR2 to induce inflammatory cascade.

In conclusion, the present *in-silico* and *in-vitro* investigations proposed a possible cross-talk between sperm and uterine epithelial cells *via* hyaluronan and hyaluronan binding receptors (CD44 and TLR2), to induce pro-inflammatory response in bovine uterus (**Figure 6**). The physiological molecule HA increase the sperm attachment to BEECs *via* CD44 which in turn activate TLR2 signaling pathway ending with transcription of the pro-inflammatory genes in response to sperm. Further investigations are required to define the specific molecule(s) from the sperm side which regulate the TLR2 to induce inflammation after sperm attachment.

## Data availability statement

The original contributions presented in the study are included in the article/**Supplementary Material**. Further inquiries can be directed to the corresponding author.

## Ethics statement

The animal study was reviewed and approved by committee on the ethics of animal experiments of the Obihiro University of Agriculture and Veterinary Medicine, Japan (Permit number 27-74).

## Author contributions

ME, AlM, IA, MY, RK, and AkM conceived and designed the experiments. ME, AlM, IA, and MY performed the experiments. ME, AlM, IA, and MY analyzed the data. AlM, RK, and AkM provided reagents/materials/analysis tools. ME, AlM, IA, MY, RK, and AM wrote the manuscript. All authors contributed to the article and approved the submitted version.
