# Supplementary material for: Hyaluronan regulates sperm-induced inflammatory response by enhancing sperm attachment to bovine endometrial epithelial cells via CD44: in-silico and in-vitro approaches
Source: Front Endocrinol (Lausanne). 2023 May 10;14:1134868. doi: 10.3389/fendo.2023.1134868 (PMC10206253; doi:10.3389/fendo.2023.1134868)
Supplement: Supplementary file 1 [file DataSheet_1.docx]

# Supplementary Data

**Details of MD simulation**

All MD simulations in this project were performed in four steps for free molecules in the water box. Some Na^+^ and Cl^−^ ions were added to neutralize the systems. In the first step, the entire system was minimized using the steepest descent algorithm and the process included 50000 cycles without any position restrains. In the second and third steps, the equilibration process was completed by the molecular dynamics of the 100 ps NVT set followed by the 100 ps NPT set by the restraining proteins, HA as 1000 kJ/mol•nm^−2^ at the harmonic force constant in the NPT phase. In the final step or production step, 150 ns MD simulations were carried out without any position restrains. The TIP3P water model was used to design the solvation box of molecules with a distance of 1.5 nm between the solute and the box walls. The simulations were performed at a temperature value of 300 K with a time step of 2 fs, taking into account the periodic boundary condition (PBC) in equilibration and production processes, and employing GROMACS 2020 using CHARMM 27 force field parameters. Topology of all ligand structures was obtained from the SwissParam website based on the CHARMM force field parameters. As for TLR2 glycoprotein, we used CHARMM-GUI to get topology and CHARMM force field parameter.

# Supplementary Figures and Tables

## Supplementary Figures


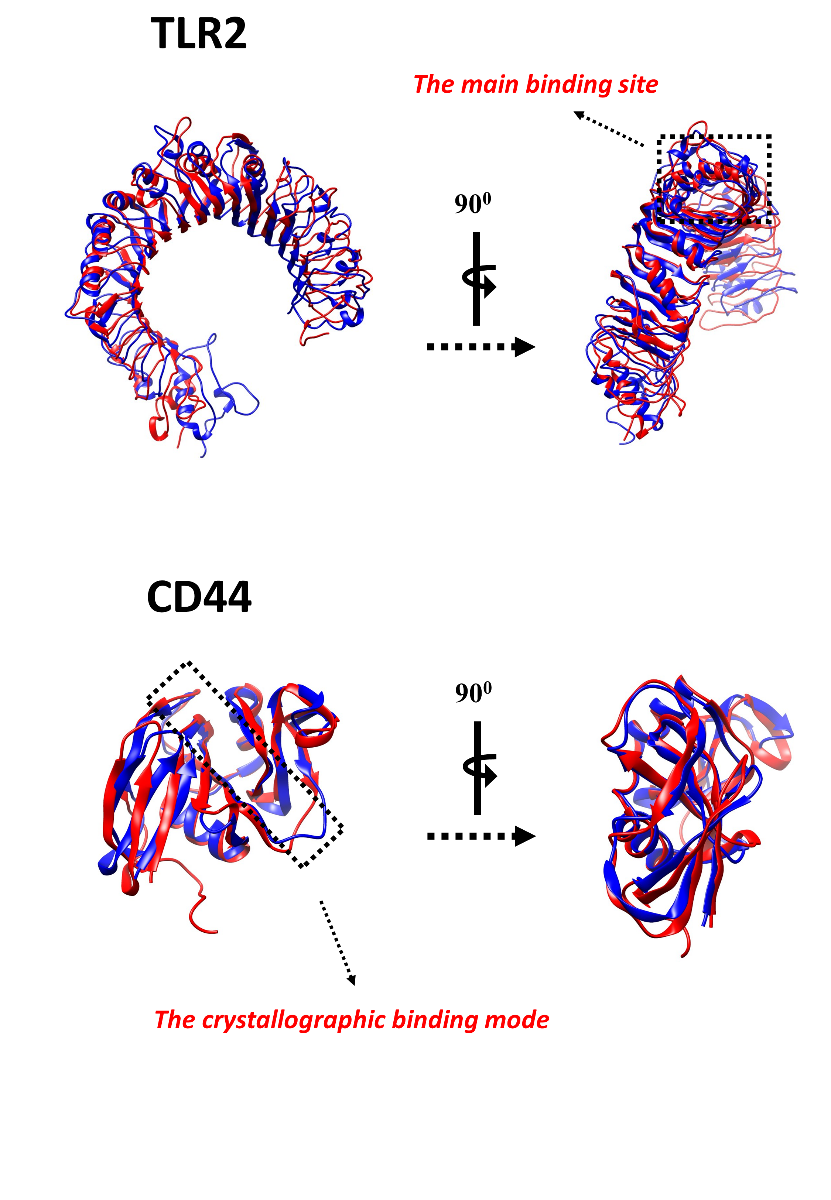


**Supplementary Figure 1.** The structural alignment of bovine (blue) and human (red) proteins using TM-align algorithms. Similar folding for TLR2 and CD44 proteins in bovine and human can be seen, as the obtained TM-score were 0.8 and 0.86 for TLR2 and CD44, respectively. Consequently, it seems that the secondary and tertiary human protein structures are nearly identical to the bovine homolog of TLR2 and CD44.


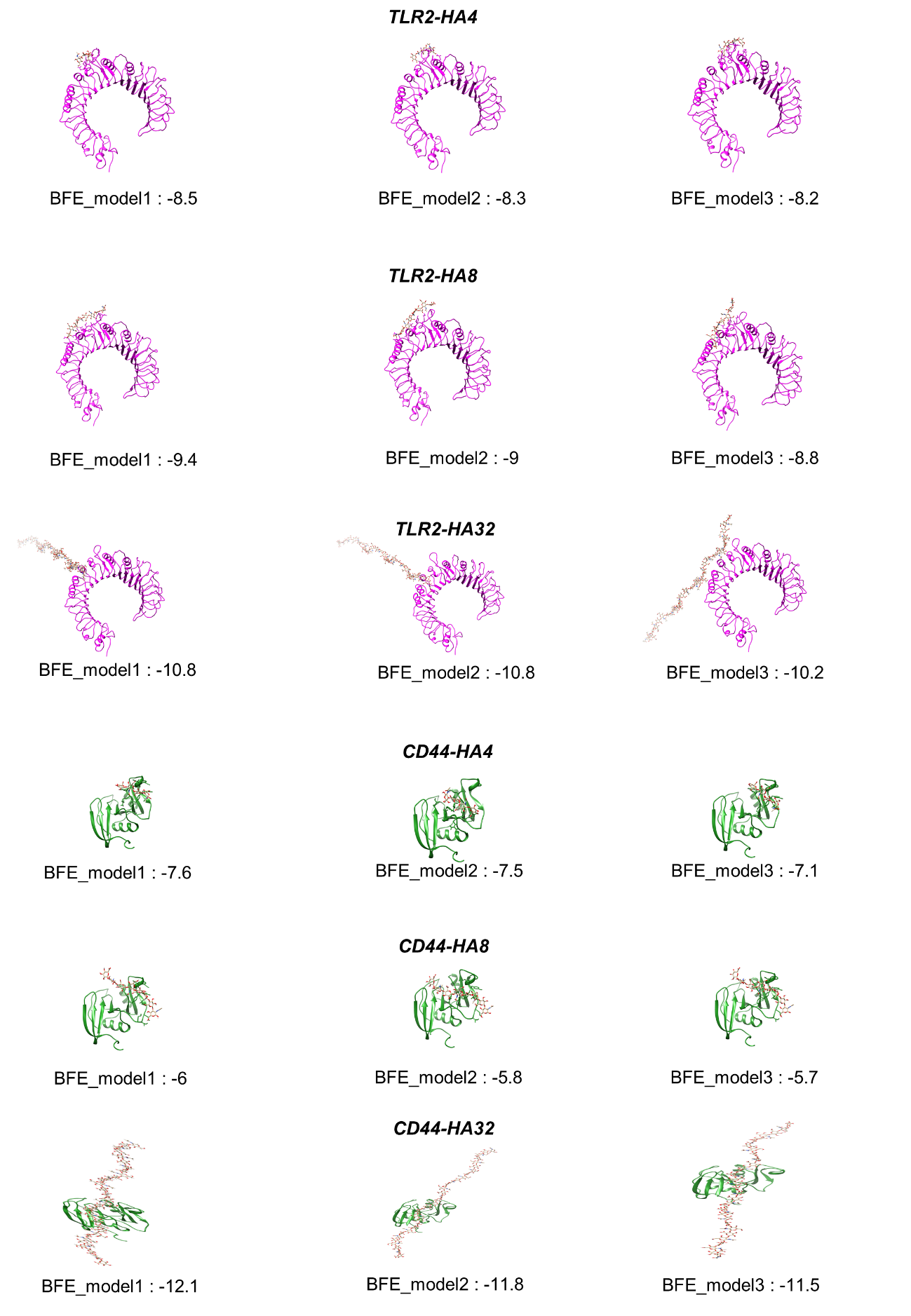


**Supplementary Figure 2.** The best docking geometries (before MD simulation phase) of complexes with predicted binding free energies.


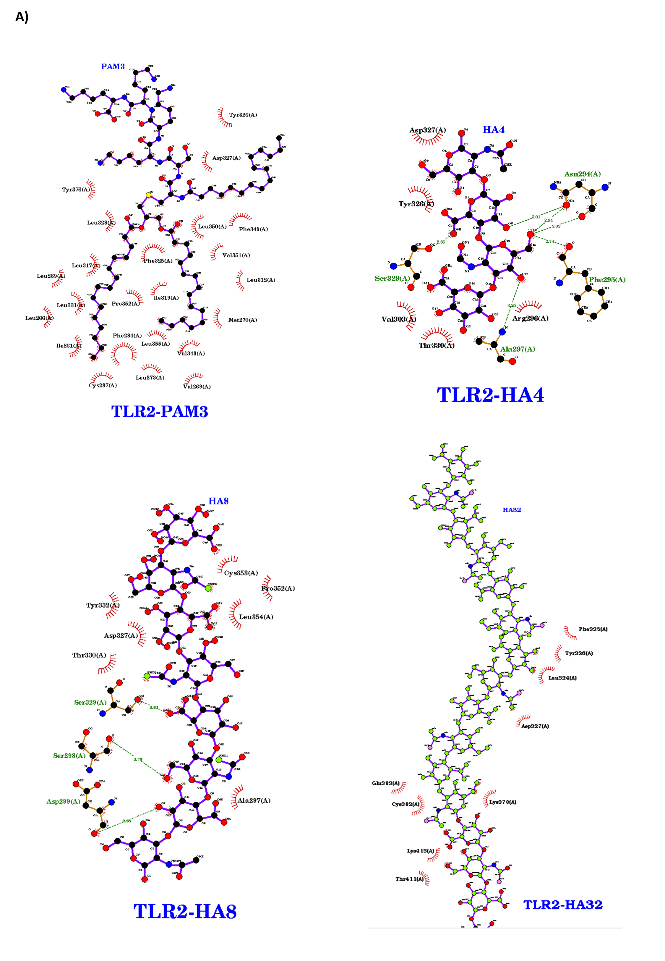


B

A


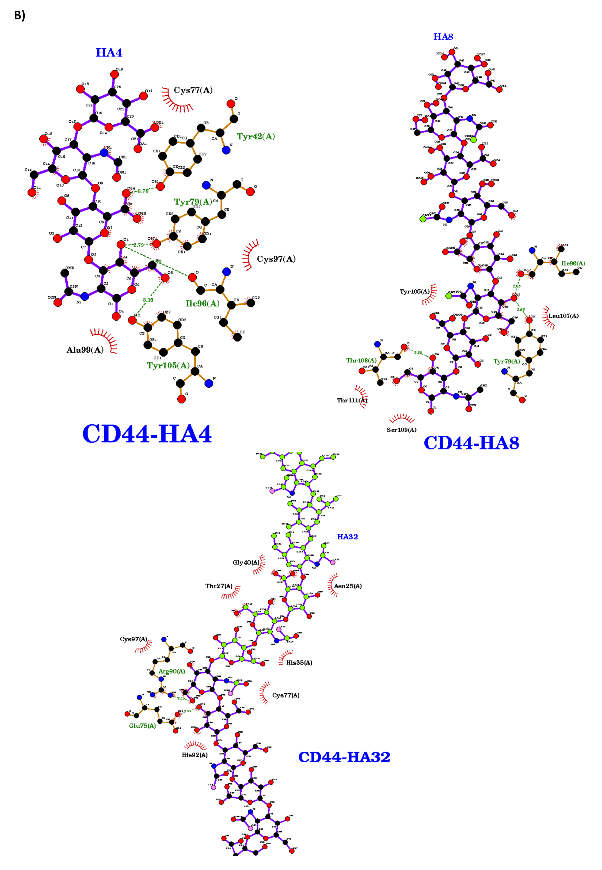


**Supplementary Figure 3.** (A) 2-D PAM3-TLR2, HA-TLR2 and (B) HA-CD44 interaction diagrams from the best docking geometries made by LIGPLOT. The spoked arcs indciate receptors generating non-covalent contacts with ligand atoms.


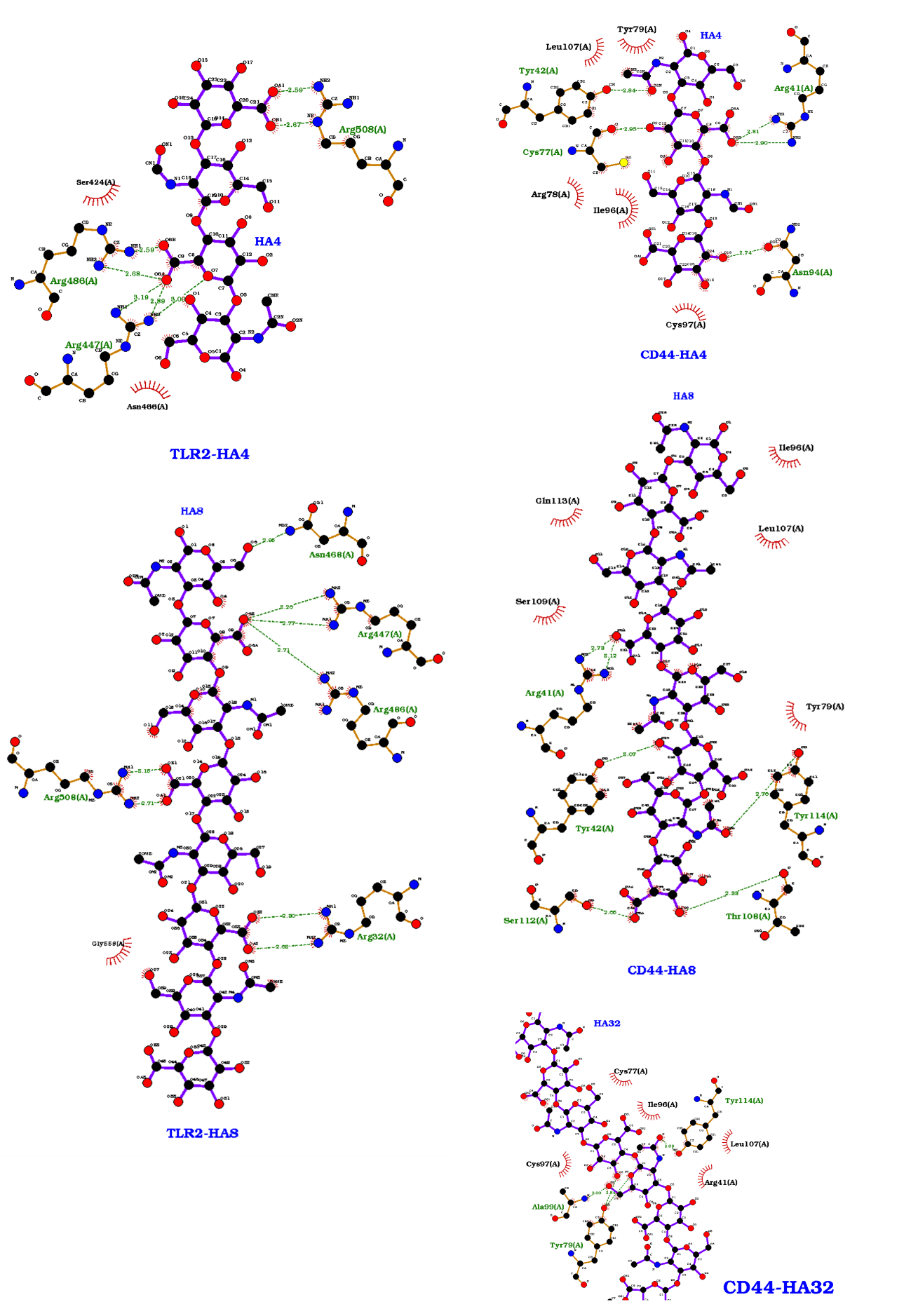


**Supplementary Figure 4.** 2-D HA-TLR2 and HA-CD44 interaction diagrams from the final snapshot of 150 ns MD simulation made by LIGPLOT. The spoked arcs indciate receptors generating non-covalent contacts with ligand atoms.


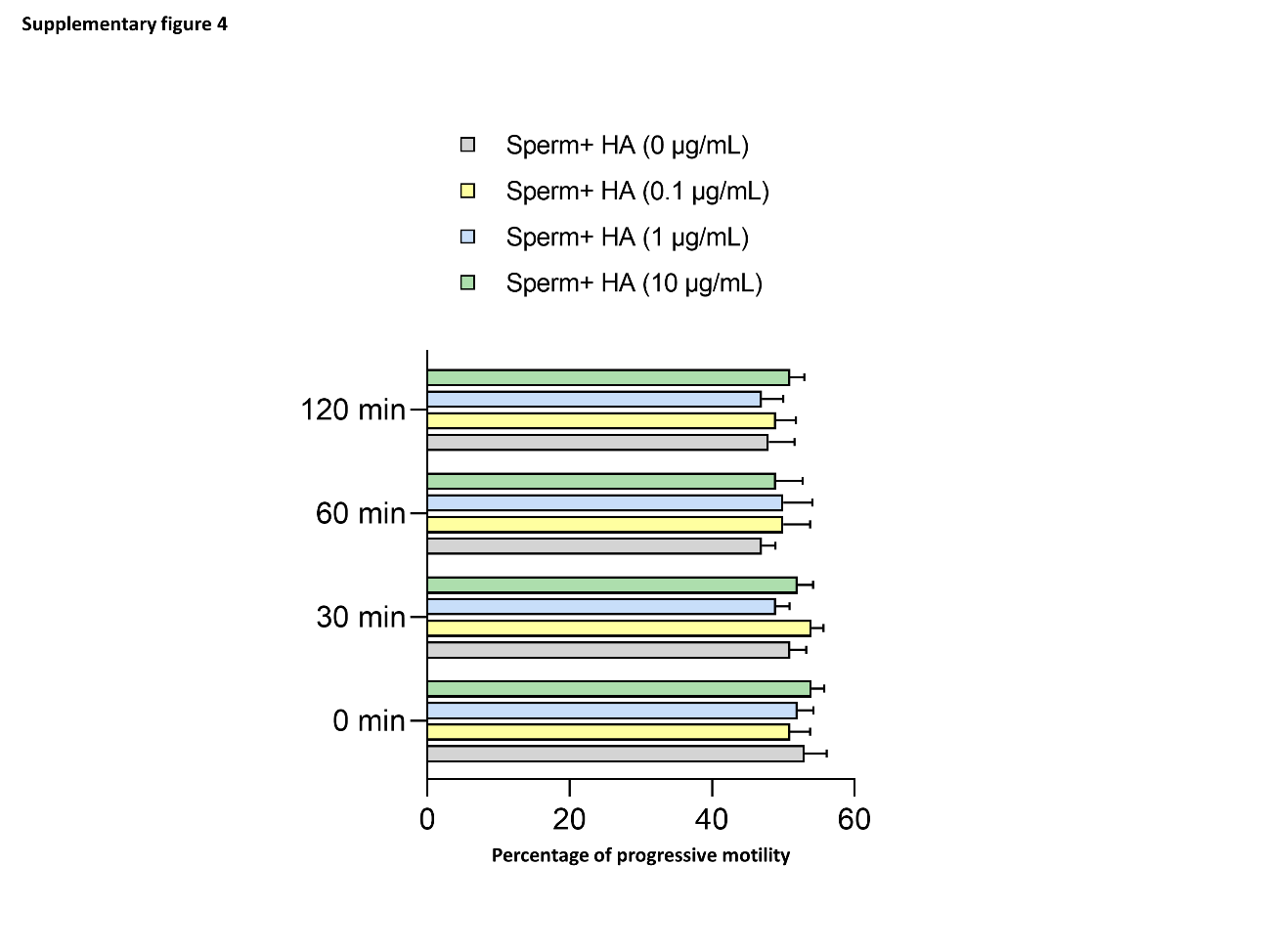


**Supplementary Figure 5.** HA has no affect on sperm motility. Sperm cells at 10^6^/ml Sp-TALP were exposed to HA (at 0, 0.1, 1, or 10 µg/mL) for 0, 30, 60, and 120 min. At each time point, progressive motility of recovered sperm from both groups was assessed and compared with those of the control group (i.e, HA at 0 µg/mL).

## Supplementary Table

**Supplementary Table 1.** List of the primers used in real-time PCR.

| ***Gene*** | **Sequence of nucleotide (5′→3′)** | | **Accession**  **no.** | **Fragment size (bp)** |
| --- | --- | --- | --- | --- |
|  | **Forward** | **Reverse** |  |  |
| *β-Actin* | TCACCAACTGGGACGACATG | CGTTGTAGAAGGTGTGGTGCC | NM_173979.3 | 51 |
| *IL8* | CCAATGGAAACGAGGTCTGC | CCTTCTGCACCCACTTTTCCT | NM_173925.2 | 51 |
| *TNFA* | CAAAAGCATGATCCGGGATG | TTCTCGGAGAGCACCTCCTC | NM_173966.3 | 51 |
| *IL1B* | AATCGAAGAAAGGCCCGTCT | ATATCCTGGCCACCTCGAAA | NM_174093.1 | 51 |
| *PGES* | AAAATGTACGTGGTGGCCGT | CTTCTTCCGCAGCCTCACTT | NM_174443.2 | 51 |
| *TLR2* | CATGGGTCTGGGCTGTCATC | CCTGGTCAGAGGCTCCTTCC | NM_174197.2 | 51 |
